# Patient-specific multimodal learning with multi-view contrastive alignment for chest X-ray report generation

**DOI:** 10.1093/bioinformatics/btag566

**Published:** 2026-07-28

**Authors:** Qiguang Miao, Kang Liu, Zhuoqi Ma, Yunan Li, Xiaolu Kang, Ruixuan Liu, Tianyi Liu, Kun Xie

**Affiliations:** School of Computer Science and Technology, Xidian University, Xi’an, Shaanxi 710071, China; Xi’an Key Laboratory of Big Data and Intelligent Vision, Xi’an, Shaanxi 710071, China; Key Laboratory of Smart Education of Shaanxi Higher Education Institutes, Xidian University, Xi’an, Shaanxi 710071, China; School of Computer Science and Technology, Xidian University, Xi’an, Shaanxi 710071, China; Xi’an Key Laboratory of Big Data and Intelligent Vision, Xi’an, Shaanxi 710071, China; Key Laboratory of Smart Education of Shaanxi Higher Education Institutes, Xidian University, Xi’an, Shaanxi 710071, China; School of Computer Science and Technology, Xidian University, Xi’an, Shaanxi 710071, China; Xi’an Key Laboratory of Big Data and Intelligent Vision, Xi’an, Shaanxi 710071, China; Key Laboratory of Smart Education of Shaanxi Higher Education Institutes, Xidian University, Xi’an, Shaanxi 710071, China; School of Computer Science and Technology, Xidian University, Xi’an, Shaanxi 710071, China; Xi’an Key Laboratory of Big Data and Intelligent Vision, Xi’an, Shaanxi 710071, China; Key Laboratory of Smart Education of Shaanxi Higher Education Institutes, Xidian University, Xi’an, Shaanxi 710071, China; School of Computer Science and Technology, Xidian University, Xi’an, Shaanxi 710071, China; Xi’an Key Laboratory of Big Data and Intelligent Vision, Xi’an, Shaanxi 710071, China; Key Laboratory of Smart Education of Shaanxi Higher Education Institutes, Xidian University, Xi’an, Shaanxi 710071, China; School of Medicine, Shanghai Jiao Tong University, Shanghai 200025, China; Department of Neurology, The First Affiliated Hospital of Harbin Medical University, Harbin, Heilongjiang 150001, China; School of Computer Science and Technology, Xidian University, Xi’an, Shaanxi 710071, China; Xi’an Key Laboratory of Big Data and Intelligent Vision, Xi’an, Shaanxi 710071, China; Key Laboratory of Smart Education of Shaanxi Higher Education Institutes, Xidian University, Xi’an, Shaanxi 710071, China

## Abstract

**Motivation:**

Radiology reports play a pivotal role in guiding treatment planning and enabling effective doctor-patient communication. However, their manual composition imposes a substantial workload on radiologists. Although automatic radiology report generation has emerged as a promising alternative, existing approaches predominantly rely on single-view chest X-rays and fail to adequately leverage patient-specific context, thereby limiting diagnostic accuracy.

**Results:**

To address this challenge, we propose *EVOKE*, a novel chest X-ray report generation framework that incorporates multi-view contrastive learning and patient-specific knowledge. Specifically, we introduce a multi-view contrastive learning method that captures semantic correspondences both among multi-view radiographs within a study and between these radiographs and their associated report, thereby improving visual representation learning. We further present a knowledge-guided report generation module that integrates available patient-specific knowledge (i.e. *indication*, which includes symptom descriptions) to facilitate the generation of accurate and coherent radiology reports. To support research in multi-view report generation, we construct Multi-view CXR and Two-view CXR datasets using publicly available sources. Our proposed EVOKE surpasses recent state-of-the-art methods across multiple datasets, achieving a 2.9% F_1_ RadGraph improvement on MIMIC-CXR, a 5.0% BLEU-1 improvement on MIMIC-ABN, a 1.5% BLEU-4 improvement on Multi-view CXR, and an 8.2% F_1,mic-14_ CheXbert improvement on Two-view CXR.

**Availability:**

Code is publicly available at https://github.com/mk-runner/EVOKE, with an archived release available on Zenodo (doi:10.5281/zenodo.21000219).

## 1 Introduction

Radiology reports (Bressem *et al*. 2021) are expert-authored clinical documents that provide structured interpretations of medical imaging examinations such as X-rays (Wang and Chen 2013, Liu *et al*. 2025b, 2026a) and CTs (Zhu *et al*. 2022, Yang *et al*. 2026). They capture observed abnormalities and preliminary diagnostic impressions, serving as a critical reference for clinical decision-making and treatment planning (Wang *et al*. 2025). However, generating radiology reports is a labor-intensive and expertise-dependent process (Liu *et al*. 2025a), imposing a substantial workload on radiologists and creating significant bottlenecks in clinical practice, particularly in resource-limited healthcare settings.

Automatic chest X-ray report generation aims to produce accurate, detailed free-text reports from radiographs, enhancing diagnostic efficiency and consistency by providing high-quality drafts for radiologists. However, single-view radiographs are often insufficient for reliable diagnosis due to projection-specific limitations and anatomical superimposition. For example, the frontal projection may provide limited characterization of retrocardiac opacities because of anatomical superimposition, obscuring findings such as hiatal hernias. Consequently, multi-view imaging protocols, including postero-anterior (PA), antero-posterior (AP), and lateral views, are routinely acquired in clinical practice to provide complementary anatomical evidence for reliable diagnosis. This multi-view setting is also reflected in publicly available datasets, such as MIMIC-CXR (Johnson *et al*. 2019) and MIMIC-ABN (Ni *et al*. 2020), which typically include a variable number of radiographs per study, an associated report, and a patient-specific *indication* (describing presenting symptoms, though occasionally missing). In clinical practice, radiologists prioritize key views and integrate supplementary images with patient symptoms to form a comprehensive assessment. In contrast, existing methods (Liu *et al*. 2024b, Tang *et al*. 2025) predominantly rely on single-view inputs, underutilizing multi-view anatomical information and neglecting patient-specific context, which often leads to inaccurate and inconsistent reports.

To leverage multi-view information, FMVP (Liu *et al*. 2024c) treats single-view images, disease labels, and medical concepts as distinct views for report generation, but does not explicitly exploit the complementary anatomical information contained in multi-view imaging. CXRMate (Nicolson *et al*. 2024) extends report generation to longitudinal multi-view settings, improving clinical accuracy while overlooking patient-specific indications. To address this limitation, Nguyen *et al*. (2023) incorporate *indications* as textual prompts; however, their approach assumes these inputs are reliable and does not account for the noise and incompleteness commonly observed in clinical records. SEI (Liu *et al*. 2024a) partially mitigates this issue via a preprocessing pipeline that removes invalid characters and normalizes gender expressions, yet it still fails to fully exploit the comprehensive anatomical information available in multi-view radiographs.

To address this challenge and emulate radiologists’ clinical practices, we propose a new framework named *EVOKE*, which incorporates multi-view images and patient-specific knowledge into the chest X-ray report generation process. More concretely, we present a multi-view contrastive learning method that utilizes a multi-view fusion module to integrate a variable number of X-rays from the same study. This method enhances visual representation by maximizing semantic correspondences both among multi-view images within a study and between these images and their corresponding report. Additionally, we introduce a knowledge-guided report generation scheme that effectively integrates available *indications* and employs a knowledge-invariant fusion module to mitigate embedding space discrepancies caused by the presence or absence of *indications*. Together, these components facilitate the generation of accurate and coherent radiology reports. We evaluate our proposed EVOKE on the MIMIC-CXR (Johnson *et al*. 2019), MIMIC-ABN (Ni *et al*. 2020), and our curated Multi-view CXR and Two-view CXR datasets. Extensive experiments confirm that EVOKE outperforms recent state-of-the-art methods. Our key contributions are as follows:

- We introduce the EVOKE framework for chest X-ray report generation, which effectively integrates multi-view images and patient-specific knowledge to enhance the accuracy of radiology reports.
- We propose a multi-view contrastive learning method that improves visual representations and accommodates variable numbers of images per study.
- We present a knowledge-guided report generation scheme that incorporates available *indications* and mitigates embedding space discrepancies caused by the presence or absence of *indications*.
- We curate Multi-view CXR and Two-view CXR datasets from two public sources, ensuring that each study includes multiple radiographs. These datasets support research on multi-view report generation, particularly in scenarios with variable or two-view setups.

## 2 Method

**Figure 1:**
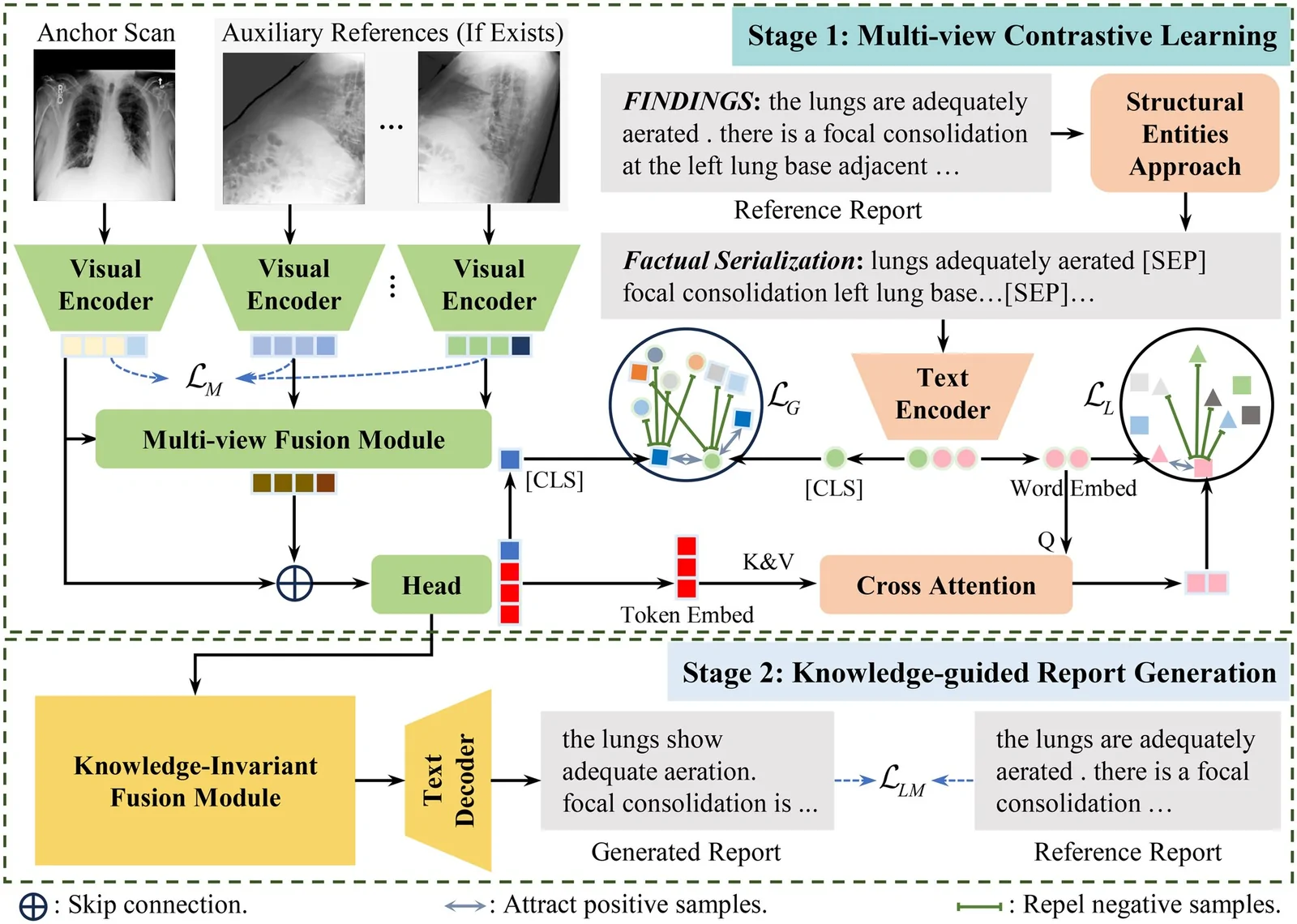
Overview of our EVOKE, comprising a visual encoder, text encoder, and text decoder. Training includes (1) multi-view contrastive representation learning and (2) knowledge-guided report generation, with only Stage 2 used at inference.

As illustrated in Fig. 1, EVOKE adopts a two-stage training pipeline. In Stage 1, we propose a multi-view contrastive learning method that captures semantic correspondences both among multi-view radiographs within a study and between these images and their associated report, thereby improving visual representation learning. In Stage 2, we present a knowledge-guided report generation scheme that flexibly incorporates patient-specific knowledge (i.e. *indications*) into the text decoder, enabling accurate and context-aware report generation.

### 2.1 Problem formulation

Let $D_{tr}={\left\{\left(x_{i,k},x_{i,\backslash k},z_{i},r_{i}\right)\right\}}_{i=1}^{n}$ be the training set, where *n* is the number of studies and $k\in [1,m_{i}]$. Each study *i* contains $m_{i}$ radiographs, an optional *indication* $z_{i}$, and a report $r_{i}$. We select a single image $x_{i,k}$ as the anchor scan for primary interpretation, while the remaining images in the same study are treated as auxiliary views $x_{i,\backslash k}=\{x_{i,j}|j\neq k,1\leq j\leq m_{i}\}$, which offer complementary anatomical context and may be empty. The *indication* $z_{i}$ describes the patient symptoms or exam rationale, offering useful contextual cues. Our goal is to learn a function $F_{\theta }\left(\cdot \right)$ that predicts the report ${\hat{r}}_{i}$ given $\left(x_{i,k},x_{i,\backslash k},z_{i}\right)$. At inference, $F_{\theta }\left(\cdot \right)$ flexibly handles arbitrary input configurations, where $x_{j,\backslash k}$ and/or $z_{j}$ may be missing, enabling adaptation to single-view, multi-view, and partial-context scenarios.

### 2.2 Multi-view contrastive learning method

#### 2.2.1 Visual features extraction

We adopt ResNet101 (He *et al*. 2016), pre-trained on ImageNet, as the visual encoder. Feature maps from its last convolutional layer are extracted as radiograph representations, denoted as $V\in R^{M\times p\times d_{1}}$. Here, $M=\sum_{i=1}^{B}m_{i}$ is the total number of radiographs in a batch of size *B*, *p* denotes the spatial resolution of the feature map, and $d_{1}$ is the channel dimension.

#### 2.2.2 Textual features extraction

Inspired by (Yan *et al*. 2023, Liu *et al.* 2026b), we adopt a structural entities approach (Liu *et al*. 2026b) to extract factual serialization from radiology reports, aiming to reduce linguistic noise for more effective cross-modal alignment. As shown in Fig. 1, factual serialization is a concise sentence constructed from clinically relevant keyword groups derived from original reports. This serialization is then fed into a six-layer pre-trained SciBERT (Beltagy *et al*. 2019), yielding textual features $T\in R^{B\times k\times d_{2}}$. Here, *k* denotes the number of textual tokens and $d_{2}$ is the token embedding dimension. These textual features T are used exclusively during Stage 1.

#### 2.2.3 Multi-positive contrastive learning between multi-view radiographs

To reinforce semantic correspondences among multi-view radiographs from the same study, we adopt multi-positive contrastive learning (Tian *et al*. 2023), which aligns radiographs within a study while differentiating them from those in unrelated studies. We first exclude studies containing only a single-view radiograph from the batch (Notably, these studies are still used for subsequent cross-modal alignment).

Given that multi-view radiographs from the same study share underlying clinical semantics, we treat all intra-study image pairs as positives and inter-study pairs as negatives. For each anchor scan $x_{i,a}$, we construct a candidate set $x_{\backslash a}$ comprising auxiliary views from the same study as well as radiographs from other studies. Let $v_{i,a}$ and $v_{\backslash a}$ denote $\ell_{2}$-normalized global visual embeddings of the anchor and candidate images, respectively. We compute a contrastive categorical distribution $q\in R^{K\times (K-1)}$ to quantify the similarity between each anchor $x_{i,a}$ and candidate set $x_{\backslash a}$:


(1) $$q_{i}=\frac{\;\text{exp}\;\left(v_{i,a}\cdot v_{\backslash a}^{T}/\tau_{1}\right)}{\sum_{j=1}^{K-1}\;\text{exp}\;\left(v_{i,a}\cdot {\left(v_{\backslash a}^{j}\right)}^{T}/\tau_{1}\right)},$$

where $K=\sum_{j=1,m_{j}>1}^{B}m_{j}$ denotes the total number of radiographs across all multi-view studies in the batch, and $\tau_{1}$ is a temperature parameter. We then define the ground-truth categorical distribution $p\in R^{K\times (K-1)}$ as:


(2) $$p_{i}=\frac{I_{\mathrm{match}}\left(x_{i,a},x_{\backslash a}\right)}{\sum_{j=1}^{K-1}I_{\mathrm{match}}\left(x_{i,a},x_{\backslash a}^{j}\right)},$$

$I_{\mathrm{match}}\left(\cdot ,\cdot \right)$ is an indicator function that returns 1 if the two radiographs belong to the same study, and 0 otherwise. The final multi-positive contrastive (MPC) loss is calculated using the cross-entropy between q and p:


(3) $$L_{M}=-\frac{1}{K}\sum_{i=1}^{K}p_{i}\;\text{log}\;q_{i}.$$

Although the number of radiographs per study varies, this variability is inherently reflected in the non-zero entries of $p_{i}$. By applying cross-entropy between $q_{i}$ and $p_{i}$, the model is encouraged to pull embeddings of all radiographs within the $i^{th}$ study closer together in the latent space, thereby enhancing intra-study semantic coherence.

#### 2.2.4 Multi-view fusion module for variable-size radio-graphs

Effective report generation requires the integration of complementary information across multiple radiographs. However, the variable number of images per study precludes straightforward feature concatenation due to inconsistent input dimensions, while simple aggregation strategies, such as average or max pooling, inevitably discard fine-grained anatomical details and inter-view dependencies.

**Figure 2:**
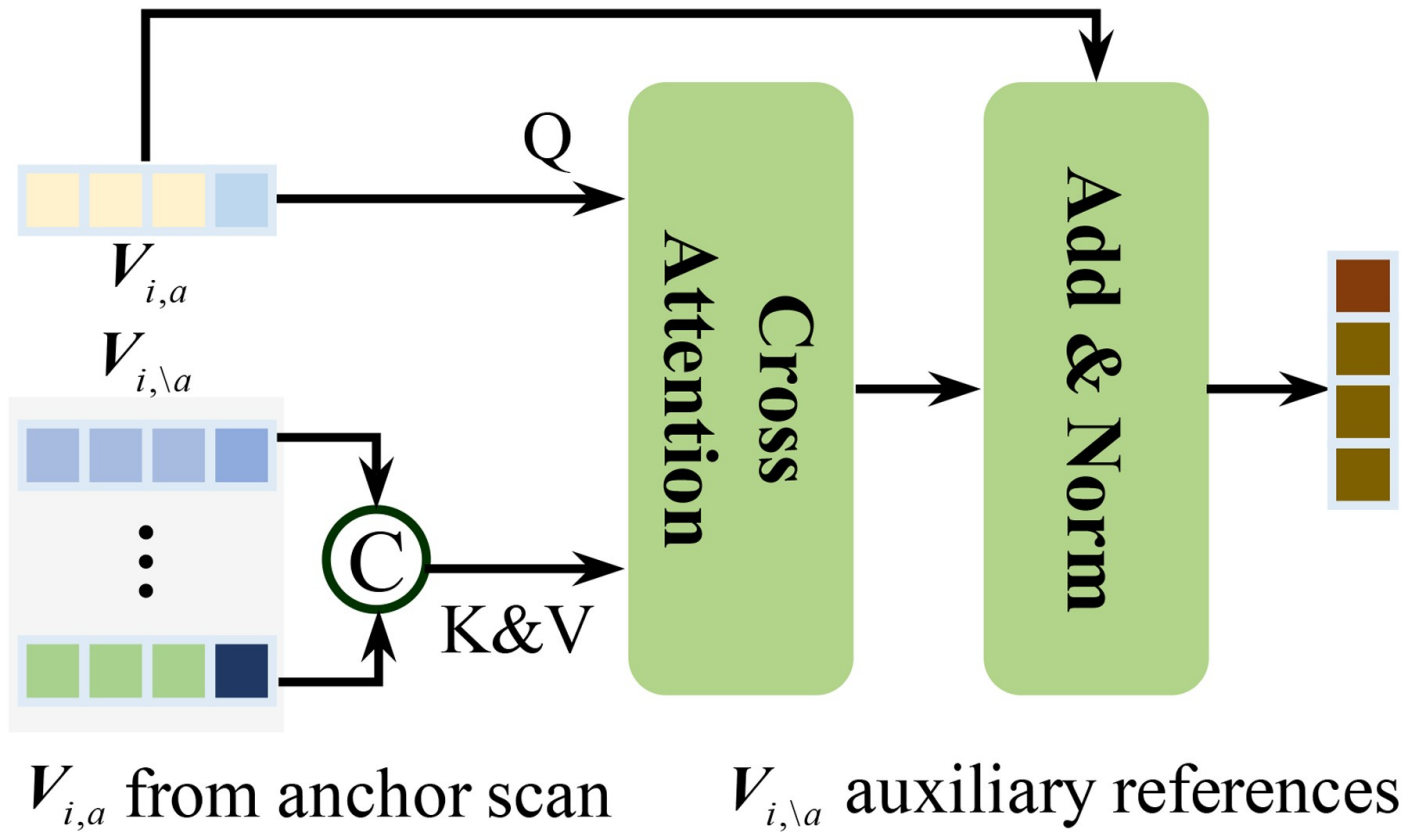
Workflow of the multi-view fusion module.

To overcome this limitation, we propose a multi-view fusion module (Fig. 2) that accommodates an arbitrary number of views via scaled dot-product cross-attention (Vaswani *et al*. 2017), denoted as ATTN(Q,K&V). Specifically, the anchor scan $x_{i,a}$ acts as queries, while auxiliary views $x_{i,\backslash a}$ serve as both keys and values. This design enables the anchor to selectively aggregate relevant contextual information, capturing inter-view dependencies. A residual connection followed by layer normalization (*LN*) preserves input semantics and stabilizes training:


(4) $${\tilde{V}}_{i,a}=LN\left(V_{i,a}+\mathrm{ATTN}\left(V_{i,a},V_{i,\backslash a}\right)\right).$$

The module operates at the study level, allowing flexible fusion over a variable number of radiographs within each study. The resulting representation $\tilde{V}=\{{\tilde{V}}_{i,a}|1\leq i\leq B\}\in R^{B\times p\times d_{1}}$ maintains consistent dimensionality while preserving contextual information from both anchor and auxiliary views.

#### 2.2.5 Instance-wise alignment loss

Drawing inspiration from (Wang *et al*. 2022, Liu *et al*. 2024a), we adopt an instance-wise alignment loss to maximize agreements between multi-view radiographs and their corresponding report in a shared latent space. We first project the fused visual features $\tilde{V}$ and textual features T into a unified latent space of dimension *d*, using a two-layer convolutional projection head. For simplicity, the projection head following the text encoder is omitted in Fig. 1. The resulting embeddings are denoted as $\bar{V}\in R^{B\times p\times d}$ for visual features and $\bar{T}\in R^{B\times k\times d}$ for textual features, where *p* and *k* are the number of tokens in visual and textual sequences.

To align multi-view images with their associated report, we construct multiple positive pairs within each study by pairing every view with the shared report. This design reflects complementary perspectives of the same study, providing diverse yet semantically consistent supervisory signals. To quantify the correspondence between visual and textual features, we compute the image-to-text contrastive categorical distribution $q^{v2t}\in R^{B\times B}$, defined as:


(5) $$q^{v2t}=\frac{\;\text{exp}\;\left(\bar{v}\cdot {\bar{t}}^{T}/\tau_{2}\right)}{\sum_{j=1}^{B}\;\text{exp}\;\left(\bar{v}\cdot {\left({\bar{t}}^{j}\right)}^{T}/\tau_{2}\right)},$$

where $\bar{v}$ and $\bar{t}$ are globally pooled and $\ell_{2}$-normalized representations of $\bar{V}$ and $\bar{T}$, and $\tau_{2}$ is a temperature parameter. A symmetric distribution $q^{t2v}$ is computed analogously for text-to-image alignment.

Since each report is paired with multiple views from the same study, we define a multi-positive ground-truth distribution $p^{g}\in R^{B\times B}$, where the probability mass is uniformly distributed among all visual features paired with the same report:


(6) $$p_{i,j}^{g}=\frac{I_{\mathrm{equal}}\left(r_{i},r_{j}\right)}{\sum_{k=1}^{B}I_{\mathrm{equal}}\left(r_{i},r_{k}\right)},$$

where $I_{\mathrm{equal}}\left(r_{i},r_{j}\right)=1$ if $r_{i}$ and $r_{j}$ are identical (i.e. originate from the same study), and 0 otherwise. The instance-wise alignment loss is expressed as:


(7) $$L_{G}=-\frac{1}{B}\sum_{i=1}^{B}\left(p_{i}^{g}\;\text{log}\;q_{i}^{v2t}+p_{i}^{g}\;\text{log}\;q_{i}^{t2v}\right).$$

$L_{G}$ treats all visual features associated with the same report as equally valid positives, encouraging consistent cross-modal representations across multiple views. Compared with prior work (Wang *et al*. 2022), our approach differs in two aspects: (1) we align images with factual serialization rather than full reports; (2) we adopt a multi-positive contrastive objective (Tian *et al*. 2023) to capture semantic correspondences between multi-view radiographs and reports, instead of relying on single-positive contrastive loss.

#### 2.2.6 Token-wise alignment loss

We adopt the token-wise alignment loss $L_{L}$ (Liu *et al*. 2026b) to enforce fine-grained correspondence between visual and textual token embeddings. Specifically, a cross-attention mechanism coupled with single-positive contrastive learning promotes token-level cross-modal alignment. As this component follows standard practice and introduces no new design, we refer readers to (Liu *et al*. 2026b) for implementation details.

To summarize, the *Stage 1 objective* is defined as:


(8) $$L_{\mathrm{pretrain}}=L_{M}+L_{G}+L_{L},$$

where $L_{M}$ enforces intra-study consistency across multiple views, $L_{G}$ promotes instance-level cross-modal alignment, and $L_{L}$ encourages token-level correspondence.

### 2.3 Knowledge-guided report generation

#### 2.3.1 Indication features extraction

**Figure 3:**
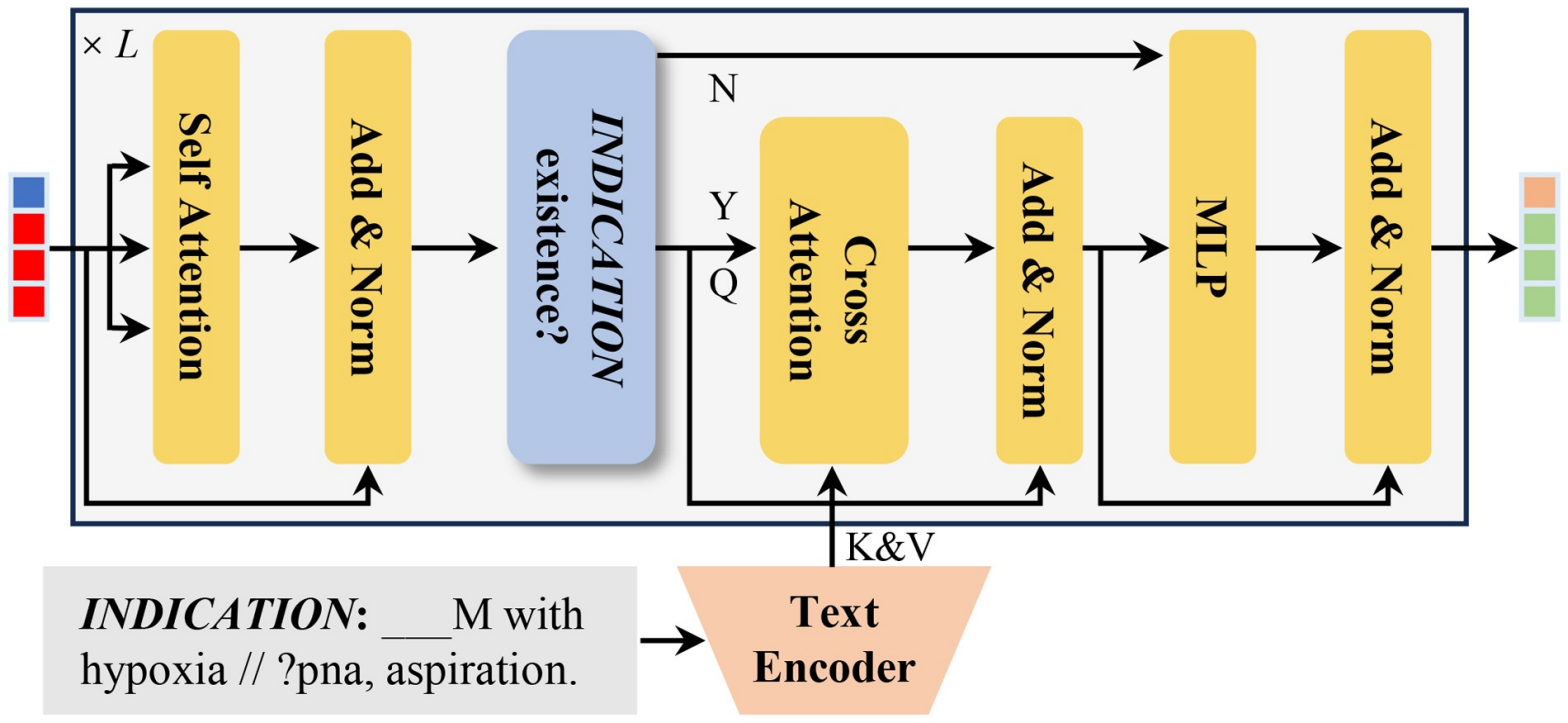
Workflow of knowledge-invariant fusion (KIF) module.

*Indications* describe patient symptoms (Fig. 3) and are collected prior to imaging. While not directly diagnostic, they provide useful context for radiograph interpretation. However, de-identification in public datasets often introduces artifacts (e.g. *___*, *@*, and *-year-old*), degrading data quality. Following (Liu *et al*. 2024a), we preprocess *indications* via noise removal and gender normalization, and encode the cleaned text using the Stage 1 initialized text encoder to obtain indication features.

#### 2.3.2 Knowledge-invariant fusion (KIF) module

As shown in Appendix Table 1, available as supplementary data at *Bioinformatics* online, *indications* are unavailable for a subset of studies. Prior methods either ignore them entirely (Wang *et al*. 2023, Shen *et al*. 2024, Liu *et al*. 2025b) or incorporate them only when available (Nguyen *et al*. 2023, Liu *et al*. 2024a), leading to inconsistent representations across samples. To mitigate this, we propose a knowledge-invariant fusion module (Fig. 3). When *indications* are present, a Transformer-decoder-style module integrates them into the visual features $\tilde{V}$ to enrich semantic representations; otherwise, a ViT-style network self-refines $\tilde{V}$. As a result, KIF unifies feature representations across studies with and without *indications*.

**Table 1:** Comparisons with SOTA methods on MIMIC-CXR (M-CXR), MIMIC-ABN (M-ABN), Multi-view CXR (MV-CXR), and Two-view CXR (TV-CXR) datasets.

Dataset	Method	Venue	Input Size	**NLG Metrics** ↑						**CE Metrics** ↑		
				B-1	B-2	B-3	B-4	MTR	R-L	RG	CX5	CX14
**Comparison with single-view methods**												
M-CXR	KiUT	CVPR’23	224	0.393	0.243	0.159	0.113	0.16	0.285	–	–	0.321
	MET	CVPR’23	–	0.386	0.25	0.169	0.124	0.152	0.291	–	–	0.311
	DMVF	MedIA’25	384	0.396	0.245	0.162	0.116	0.154	0.289	–	–	0.413
	R2Gen	EMNLP’20	224	0.353	0.218	0.145	0.103	0.142	0.277	0.207	0.34	0.34
	CMN	ACL’21	224	0.353	0.218	0.148	0.106	0.142	0.278	0.22	0.461	0.391
	MAN	AAAI’24	224	0.396	0.244	0.162	0.115	0.151	0.274	–	–	0.389
	DACG	MedIA’25	224	0.398	0.249	0.167	0.117	0.162	0.29	–	–	0.389
	SA	EMNLP’23	256	–	0.184	–	–	–	–	0.228	–	0.394
	GPT-4V	–	–	0.164	–	–	–	–	0.132	–	0.258	0.355
	LLaVA-Med	NIPS’23	–	0.354	–	–	0.149	–	–	0.191	0.439	0.427
	Med-LLM	ACMMM’24	224	–	–	–	0.128	0.161	0.289	–	–	0.395
	PMRG	AAAI’24	224	0.398	–	–	0.112	0.157	0.268	–	–	0.476
	SEI	MICCAI’24	224	0.382	0.247	0.177	0.135	0.158	0.299	0.249	0.542	0.46
	RRG-Mamba	IJCAI’25	224	0.406	0.253	0.169	0.121	0.154	0.293	–	–	0.475
	MambaGen	ESWA’26	224	0.405	0.252	0.169	0.127	0.155	0.292	–	–	0.392
	CXRMate	IMU’24	384	0.361	0.223	0.15	0.108	0.159	0.263	0.238	–	0.422
	FMVP	TMM’24	224	0.389	0.236	0.156	0.108	0.15	0.284	–	–	0.336
	**EVOKE**	Ours	224	0.395	0.262	0.19	0.147	0.167	0.311	0.276	0.557	0.499
	**EVOKE**	Ours	384	**0.408**	**0.271**	**0.197**	**0.151**	**0.171**	**0.313**	**0.278**	**0.578**	**0.517**
	Δ delta	–	–	0.20%	1.80%	2.00%	0.20%	0.90%	1.40%	2.90%	3.60%	4.10%
M-ABN	R2Gen ^♯^	EMNLP’20	224	0.253	0.144	0.092	0.063	0.106	0.229	0.179	0.501	0.442
	CMN^♯^	ACL’21	224	0.256	0.147	0.095	0.066	0.11	0.23	0.183	0.528	0.46
	SEI^♯^	MICCAI’24	224	0.279	0.167	0.112	0.079	0.117	0.237	0.2	0.529	0.454
	**EVOKE**	Ours	224	0.310	0.185	0.125	0.09	0.127	0.246	0.214	0.535	0.482
	**EVOKE**	Ours	384	**0.329**	**0.196**	**0.131**	**0.093**	**0.134**	**0.255**	**0.22**	**0.545**	**0.503**
	Δ ↑	–	–	5.00%	2.90%	1.90%	1.40%	1.70%	1.80%	2.00%	1.60%	3.70%
MV-CXR	R2Gen^♯^	EMNLP’20	224	0.359	0.225	0.155	0.114	0.143	0.297	0.255	0.431	0.384
	CMN^♯^	ACL’21	224	0.404	0.252	0.17	0.122	0.16	0.311	0.279	0.475	0.416
	SEI^♯^	MICCAI’24	224	0.406	0.26	0.184	0.138	0.166	0.317	0.294	0.524	0.463
	**EVOKE**	Ours	224	0.413	0.274	0.199	0.152	0.174	0.335	0.328	0.515	0.487
	**EVOKE**	Ours	384	**0.415**	**0.276**	**0.2**	**0.153**	**0.177**	**0.336**	**0.329**	**0.557**	**0.508**
	Δ ↑	–	–	0.90%	1.60%	1.60%	1.50%	1.10%	1.90%	3.50%	3.30%	4.50%
**Comparison with two-view methods**												
TV-CXR	R2Gen^♯^	EMNLP’20	224	0.346	0.219	0.153	0.113	0.141	0.302	0.267	0.413	0.4
	CMN^♯^	ACL’21	224	0.387	0.241	0.166	0.122	0.151	0.31	0.268	0.437	0.425
	**EVOKE**	Ours	224	0.393	0.256	0.184	0.14	0.165	0.322	0.304	0.531	0.501
	**EVOKE**	Ours	384	**0.411**	**0.27**	**0.195**	**0.15**	**0.172**	**0.326**	**0.302**	**0.547**	**0.507**
	Δ ↑	–	–	2.40%	2.90%	2.90%	2.80%	2.10%	1.60%	3.60%	11.00%	8.20%

Δ denotes EVOKE's improvement over the strongest baseline. ^♯^Indicates results reproduced from official codes; others are taken from the original papers. Best and second-best results are highlighted in bold and blue, respectively.

#### 2.3.3 Report generation

Our text decoder adopts the memory-driven Transformer (Chen *et al*. 2020). To enhance clinical context awareness, we employ KIF module to incorporate available *indications* into the visual features, yielding semantically enriched representations. These fused features are then fed into the decoder, which generates reports in an auto-regressive manner. Stage 2 is optimized using the negative log-likelihood loss:


(9) $$L_{LM_{i}}=-\sum_{t=1}^{M}\;\text{log}\;P\left({\hat{w}}_{i}^{t}|x_{i},z_{i},{\hat{w}}_{i}^{j<t}\right),$$

where ${\hat{w}}_{i}^{t}$ denotes the token generated at step *t* for the $i^{th}$ study, $x_{i}$ the multi-view radiographs, and $z_{i}$ the associated *indication* (if available). *M* is the maximum number of generated tokens.

## 3 Results

### 3.1 Experimental setup

#### 3.1.1 Datasets

*(1) MIMIC-CXR* (Johnson *et al*. 2019), a large-scale chest X-ray dataset with free-text reports and a variable number of radiographs per study; *(2) MIMIC-ABN* (Ni *et al*. 2020), a subset of MIMIC-CXR focusing on abnormal report sentences; *(3) Multi-view CXR*, our curated multi-view dataset constructed from MIMIC-CXR and IU X-ray (Demner-Fushman *et al*. 2016); *(4) Our Two-view CXR*, a variant of Multi-view CXR restricted to exactly two views per study. Following (Chen *et al*. 2020, Wang *et al*. 2023), we treat the *FINDINGS* section as the reference report. MIMIC-CXR and MIMIC-ABN adopt their official splits. For Multi-view CXR, we follow the MIMIC-CXR split for its MIMIC portion and the standard split (Chen *et al*. 2020, Shen *et al*. 2024) for the IU X-ray subset. Dataset statistics are provided in Appendix Table 1, available as supplementary data at *Bioinformatics* online.

#### 3.1.2 Evaluation metrics

We evaluate performance using both natural language generation (NLG) and clinical efficacy (CE) metrics. NLG metrics measure the lexical similarities between generated and reference reports, including BLEU-*n* (B-*n*), METEOR (MTR), and ROUGE-L (R-L). CE metrics evaluate clinical correctness, including F_1_ RadGraph (RG) (Jain *et al*. 2021) and CheXbert-based F_1_ scores on 5 and 14 observations (CX5, CX14) (Smit *et al*. 2020). Specifically, RG quantifies overlap in clinical entities and their relations, while CX5 and CX14 evaluate the model’s ability to identify key clinical observations (e.g. *Edema*, *Cardiomegaly*) via micro-averaged F_1_ in a multi-label setting.

#### 3.1.3 Implementation details

The batch size is set to 32. We fix $\tau_{1}=\tau_{2}=0.5$ and use a single block in the knowledge-invariant fusion (KIF) module. The maximum generation length *M* is set to 100 across all datasets. Model selection is based on a composite validation score (B-4, RG, and CX14), and final results are reported on the test set. Additional details are provided in the Appendix, available as supplementary data at *Bioinformatics* online.

### 3.2 Quantitative evaluation

#### 3.2.1 Comparison with state-of-the-art (SOTA) methods

We compare EVOKE with 17 SOTA methods spanning diverse paradigms: knowledge graph-based models (KiUT (Huang *et al*. 2023), MET (Wang *et al*. 2023)), contrastive learning (DMVF (Tang *et al*. 2025)), memory-based methods (R2Gen (Chen *et al*. 2020), CMN (Chen *et al*. 2021), MAN (Shen *et al*. 2024), DACG (Lang *et al*. 2025)), LLM-based methods (SA (Yan *et al*. 2023), GPT-4V (OpenAI 2023), LLaVA-Med (Li *et al*. 2023), Med-LLM (Liu *et al*. 2024b), PMRG (Jin *et al*. 2024)), Mamba-based methods (RRG-Mamba (Hou *et al*. 2025), MambaGen (Hou *et al*. 2026)), context-aware model (SEI (Liu *et al*. 2024a)), and multi-view approaches (CXRMate (Nicolson *et al*. 2024), FMVP (Liu *et al*. 2024c)).

Results are presented in Table 1, where “*Input Size*” denotes the image resolution (e.g. 224 for 224×224). EVOKE consistently outperforms all baselines across four datasets, achieving gains of +2.9% RG on MIMIC-CXR, +5.0% B-1 on MIMIC-ABN, +1.5% B-4 on Multi-view CXR, and +8.2% CX14 on Two-view CXR. These improvements demonstrate its effectiveness in generating clinically accurate reports. Increasing the input resolution to 384×384 improves performance on most metrics, highlighting the benefit of higher-resolution inputs.

#### 3.2.2 Clinical accuracy of 14 observations

Appendix Table 2, available as supplementary data at *Bioinformatics* online reports performance on 14 thoracic observations for MIMIC-CXR and Two-view CXR. EVOKE significantly improves F_1_ over R2Gen (Chen *et al*. 2020) across most categories. Notably, despite not being tailored for class imbalance, EVOKE improves F_1_ for rare findings such as *Pleural Other* and *Fracture* by 12% and 2.3% in MIMIC-CXR, and by 1.7% and 7% in Two-view CXR, respectively.

**Table 2:** Ablation study of Stage 1 on MIMIC-CXR with input resolution 224×224.

Model	**Stage 1**				**NLG Metrics** ↑				**CE Metrics** ↑		
	F/R	$L_{M}$	$L_{G}$	$L_{L}$	B-2	B-4	MTR	R-L	RG	CX5	CX14
w/o Stage 1	–	✗	✗	✗	0.247	0.134	0.158	0.296	0.252	0.545	0.480
w/o $L_{L}$	F	✓	✓	✗	**0.263**	0.145	0.166	0.305	0.268	0.540	0.480
w/o $L_{G}$	F	✓	✗	✓	0.246	0.136	0.157	0.305	0.255	0.513	0.450
w/o $L_{M}$	F	✗	✓	✓	0.252	0.142	0.162	0.307	0.266	0.536	0.477
w/o FS	R	✓	✓	✓	0.258	0.142	0.164	0.307	0.266	0.522	0.470
**EVOKE (Ours)**	F	✓	✓	✓	0.262	**0.147**	**0.167**	**0.311**	**0.276**	**0.557**	**0.499**

“F/R” indicates the use of **F**actual serialization or full **R**eport for cross-modal alignment. **Best values** are highlighted in **bold**.

#### 3.2.3 Evaluation via large language models

**Figure 4:**
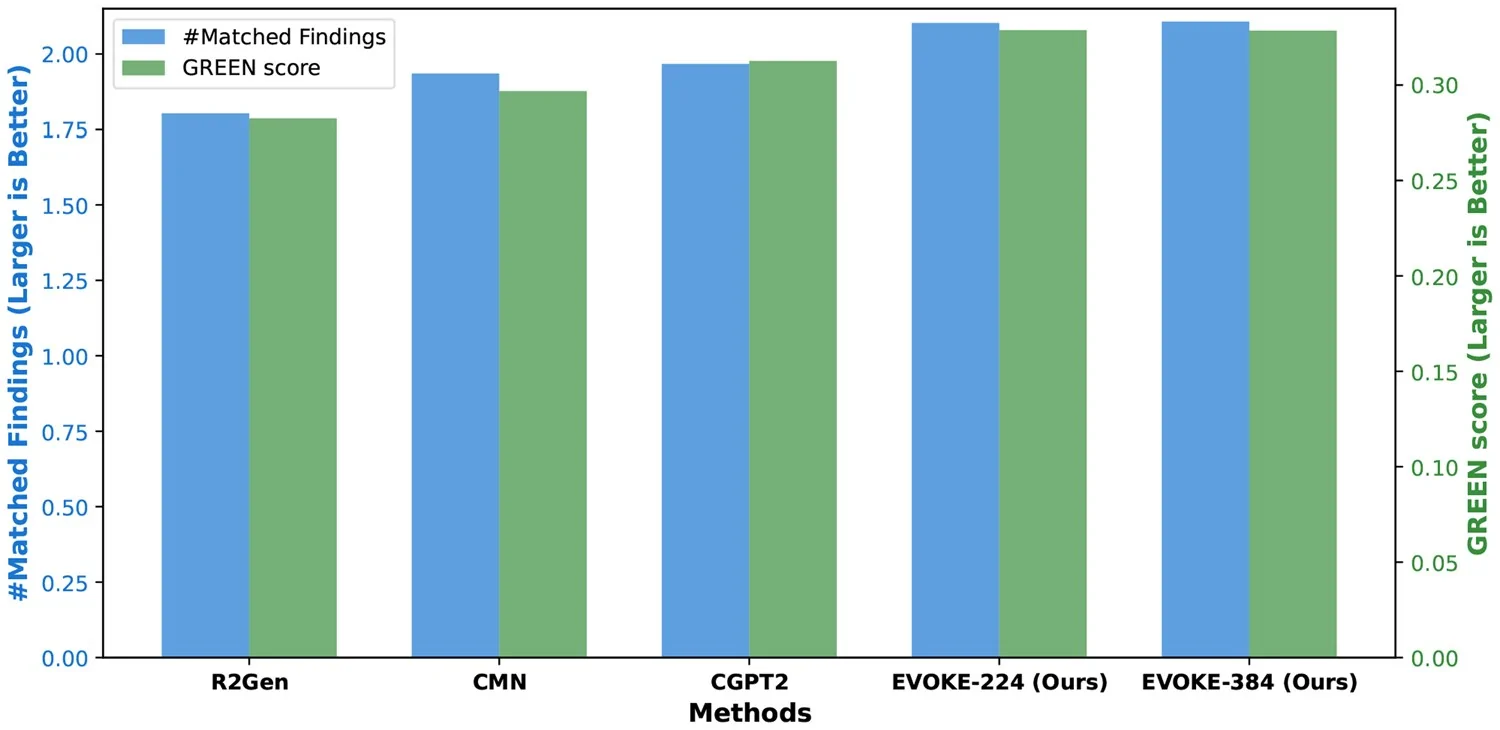
Comparisons with baselines on MIMIC-CXR dataset using *#Matched Findings* and *GREEN score*. “EVOKE-s” denotes a resolution-specific variant of our EVOKE.

We further assess clinical quality using GREEN (Ostmeier *et al*. 2024), a fine-tuned LLaMA-2 model for detecting clinically significant errors in radiology reports. As shown in Fig. 4, we compare EVOKE with R2Gen (Chen *et al*. 2020), CMN (Chen *et al*. 2021), and CGPT2 (Nicolson *et al*. 2023) on MIMIC-CXR. EVOKE consistently achieves the best performance in both *#Matched Findings* and *GREEN score*, demonstrating its superior clinical accuracy and reliability.

### 3.3 Ablation study

#### 3.3.1 Effect of multi-view contrastive learning (stage 1)

We evaluate Stage 1 on MIMIC-CXR (Table 2). Removing any components—multi-positive contrastive loss $L_{M}$, instance-wise alignment $L_{G}$, or token-wise alignment $L_{L}$—consistently degrades both NLG and CE metrics, indicating their complementary roles. Replacing factual serialization with full reports for cross-modal alignment further reduces performance. Overall, EVOKE surpasses all ablated variants, confirming the critical role of Stage 1 in improving both generation quality and clinical relevance.

#### 3.3.2 Effect of knowledge-guided report generation (stage 2)

We evaluate Stage 2 on the MIMIC-CXR dataset (Table 3). Removing Stage 2 yields the lowest performance across all NLG and CE metrics, underscoring its necessity. Incorporating *indications* without KIF (w/o KIF) provides modest gains but remains suboptimal, suggesting that latent space misalignment hinders effective use of patient-specific knowledge. In contrast, the full EVOKE model—integrating both *indications* and the KIF module—consistently achieves the best results, particularly on clinical metrics (e.g. CX14 reaches 0.499). These findings highlight the importance of leveraging patient-specific knowledge and validate the effectiveness of the knowledge-invariant fusion module.

**Table 3:** Ablation study of Stage 2 on MIMIC-CXR with input resolution 224×224.

Model	Stage 2		**NLG Metrics** ↑				**CE Metrics** ↑		
	Indications	KIF	B-2	B-4	MTR	R-L	RG	CX5	CX14
w/o Stage 2	✗	✗	0.226	0.108	0.148	0.279	0.230	0.539	0.477
w/o KIF	✓	✗	0.259	0.145	0.164	**0.312**	0.272	0.548	0.488
**EVOKE (Ours)**	✓	✓	**0.262**	**0.147**	**0.167**	0.311	**0.276**	**0.557**	**0.499**

“KIF” denotes the knowledge-invariant fusion module. “w/o KIF” represents conditionally incorporating *indications* only when available, leading to representation inconsistencies. **Best values** are highlighted in **bold**.

#### 3.3.3 Effect of indications

To investigate the contribution of *indications*, we conducted experiments on the MIMIC-CXR subset in which all studies contain indication (n = 2225). As shown in Table 4, removing *indications* markedly degrades performance across all metrics, reducing BLEU-4 from 0.166 to 0.085 and F_1_ RadGraph from 0.302 to 0.222. The degradation is particularly pronounced on the clinical metrics (RG, CX5, and CX14), indicating that *indications* provide important contextual cues for clinically accurate report generation. Using raw *indications* consistently improves performance over the indication-free setting, whereas progressively corrupting the *indications* leads to steady performance degradation. When all *indications* are corrupted, performance deteriorates substantially, suggesting that severely mismatched clinical context can mislead report generation. Compared with the raw-indication baseline, EVOKE achieves the best overall performance, improving BLEU-4 and F1 RadGraph by 3.1 and 1.5 percentage points, respectively. These results demonstrate that effectively modeling clinical *indications* is essential for generating accurate and clinically faithful radiology reports.

**Table 4:** Effect of *indications* on report generation at a resolution of 384×384.

Settings	**B-2** ↑	**B-4** ↑	**MTR** ↑	**R-L** ↑	**RG** ↑	**CX5** ↑	**CX14** ↑	**#Matched Findings** ↑	**GREEN** ↑
w/o Indication	0.200	0.085	0.138	0.255	0.222	0.562	0.496	1.785	0.271
Raw	0.261	0.135	0.165	0.296	0.287	0.562	0.501	2.208	0.328
Random-10%	0.285	0.160	0.177	0.315	0.297	0.575	0.511	2.329	0.344
Random-50%	0.254	0.133	0.161	0.291	0.273	0.557	0.495	2.198	0.323
Random-100%	0.215	0.093	0.144	0.259	0.242	0.537	0.471	1.983	0.295
**EVOKE**	**0.292**	**0.166**	**0.180**	**0.321**	**0.302**	**0.577**	**0.514**	**2.362**	**0.349**

Experiments are conducted on the MIMIC-CXR subset in which all studies contain *indications* (n=2225). *Random-K%* denotes that K% of the test samples are randomly selected, and their *indications* are replaced by *indications* sampled from the training set. **Best values** are highlighted in **bold**.

#### 3.3.4 Effect of multi-view radiographs

To assess the benefit of multi-view radiographs, we construct a subset of the MIMIC-CXR test set containing only studies with three available radiographs (n=237). As shown in Table 5, increasing the number of views consistently improves both lexical and clinical metrics. Specifically, BLEU-4 improves from 0.099 to 0.151, while RG and CX5 increase from 0.251 to 0.282 and from 0.535 to 0.570, respectively. These results suggest that additional views provide complementary diagnostic information that cannot be reliably inferred from a single projection. Importantly, the performance gains come at minimal computational cost. Using three views requires less than 3 GB of GPU memory and only 0.300 s per study, enabling a throughput of over three studies per second. This favorable accuracy-efficiency trade-off makes EVOKE practical for real-world clinical deployment.

**Table 5:** Effect of multi-view images on report generation at a resolution of 384×384.

#Views	**B-2** ↑	**B-4** ↑	**RG** ↑	**CX5** ↑	**CX14** ↑	**#Matched Findings** ↑	**GREEN** ↑	**GPU Memory** ↓	**Inference Time** ↓
1	0.200	0.099	0.251	0.535	0.416	1.992	0.304	2.338 GB	0.278 s/sample
2	0.271	0.147	0.277	0.566	0.468	2.325	0.341	2.323 GB	0.290 s/sample
3	**0.273**	**0.151**	**0.282**	**0.570**	**0.464**	**2.418**	**0.353**	2.365 GB	0.300 s/sample

*#Views* denotes the number of radiographic views. **Best values** are highlighted in **bold**.

#### 3.3.5 Model benefits from multi-view radiographs and *indications*

Table 6 reports performance on MIMIC-CXR subsets stratified by the presence of multi-view radiographs and *indications*. Both factors yield consistent gains in NLG and RG metrics when available. This indicates that multi-view images provide complementary anatomical information, while *indications* offer critical clinical context—together enabling more accurate and context-aware report generation.

**Table 6:** Breakdown of EVOKE performance on MIMIC-CXR test set at 224×224, stratified by: (1) the multi-view availability (MV), and (2) the presence of *indications* (Ind).

Metric	w/ MV	w/o MV	w/ Ind	w/o Ind
%	70.7	29.3	57.8	42.2
B-1 ↑	**0.406**	0.369	**0.413**	0.372
B-2 ↑	**0.270**	0.242	**0.280**	0.238
B-3 ↑	**0.196**	0.176	**0.206**	0.169
B-4 ↑	**0.151**	0.136	**0.161**	0.128
MTR ↑	**0.170**	0.157	**0.174**	0.157
R-L ↑	**0.316**	0.298	**0.321**	0.298
RG ↑	**0.287**	0.249	**0.299**	0.244

**Best values** are highlighted in **bold**.

### 3.4 Qualitative results

**Figure 5:**
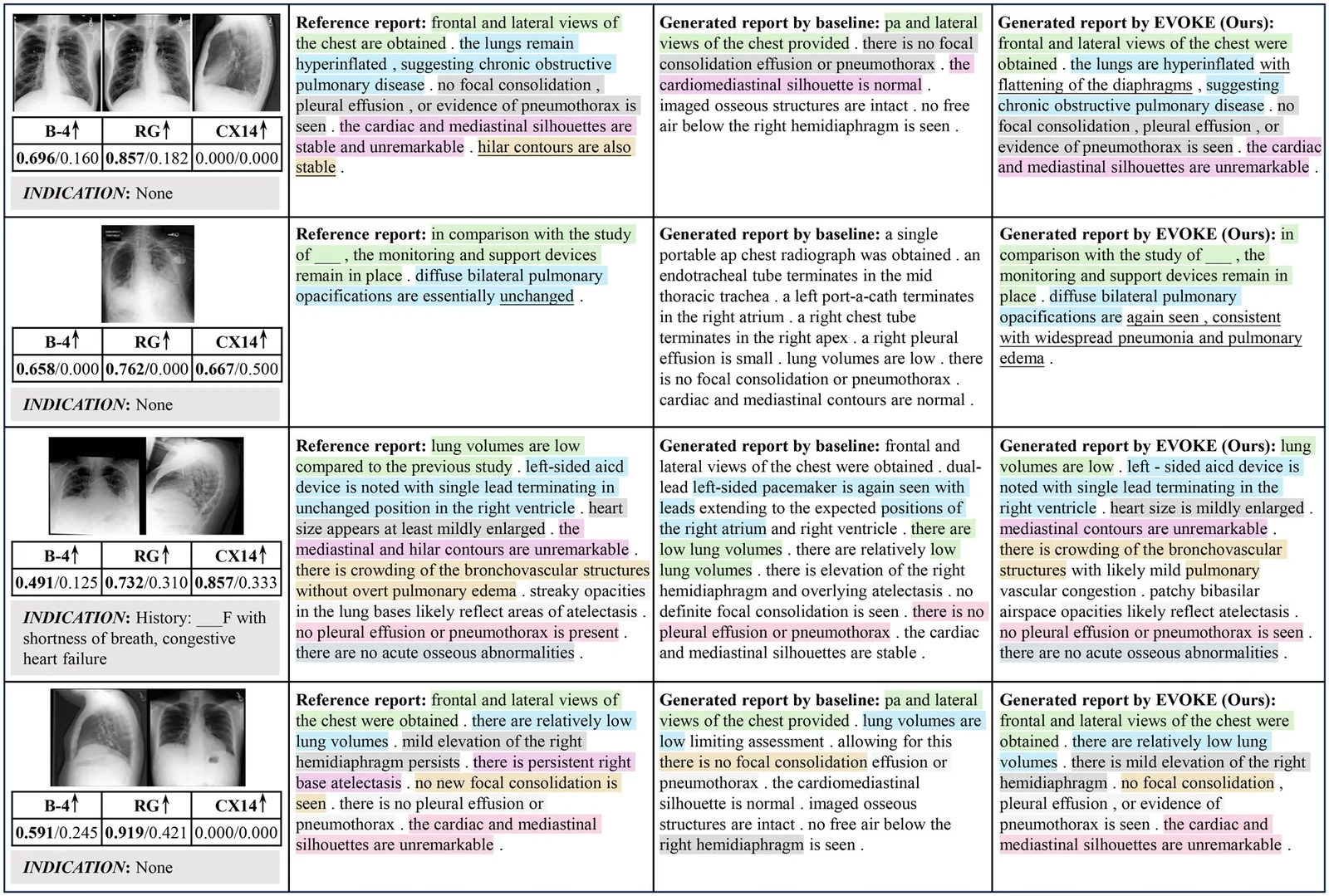
Qualitative examples of generated reports from the MIMIC-CXR test set at 224×224. Each cell “A/B” reports scores for EVOKE and baseline (Chen *et al*. 2020), respectively. Reference sentences are color-coded, with corresponding generated sentences highlighted in matching colors. Erroneous sentences are underlined.

As illustrated in Fig. 5, EVOKE consistently outperforms the baseline (Chen *et al*. 2020) across diverse input conditions, including multi-view (two or three images), single-view, and scenarios with or without *indications*. *(1) Multi-view input enhances performance:* In Cases 1 and 4, EVOKE effectively integrates frontal and lateral images to identify critical findings such as lung hyperinflation (Case 1) and diaphragmatic elevation (Case 4), producing reports that closely align with the reference. In contrast, the baseline tends to generate generic descriptions that omit key diagnostic details. Additional qualitative examples are presented in Appendix Fig. 1, available as supplementary data at *Bioinformatics* online. *(2)* Indication *improves contextual understanding:* In Case 3, incorporating clinical context enables EVOKE to more accurately characterize heart enlargement, pulmonary vascular congestion, and device positioning. *(3) Robustness in single-view settings:* In Case 2, despite the presence of only a single image and the absence of *indication*, EVOKE still generates a coherent and clinically consistent report. Overall, these results empirically demonstrate that EVOKE generalizes well to diverse real-world clinical scenarios, including single-view, multi-view, and partial-context settings.

Despite these gains, EVOKE remains sensitive to subtle or ambiguous findings. For instance, in Case 3, it predicts *mild pulmonary vascular congestion* while the reference reports *no overt pulmonary edema*, suggesting over-interpretation of uncertain cues. This behavior may arise from limited uncertainty modeling and an over-reliance on co-occurrence patterns. Future work will investigate calibrated uncertainty estimation and counterfactual training strategies to encourage more conservative, clinically aligned predictions in such scenarios.

### 3.5 Human evaluation

To objectively evaluate the quality of generated reports, we conduct a double-blind human study with two experienced radiologists. We randomly sample 50 cases from the MIMIC-CXR test set, each consisting of a reference report, multi-view radiographs, and three candidate reports generated by R2Gen (Chen *et al*. 2020), CMN (Chen *et al*. 2021), and our EVOKE-224, presented in random order. Evaluation covers two aspects: *(1) Error Severity Analysis:* Each report is examined for four error types: (I) false prediction of findings, (II) omission of findings, (III) incorrect location or position, and (IV) incorrect severity. Errors are further categorized into three levels: significant error (SE), insignificant error (IE), and no error (NE). *(2) Overall Quality Ranking:* For each case, radiologists rank the three reports as best, moderate, or worst based on clinical accuracy and coherence.

As summarized in Table 7, EVOKE consistently outperforms both R2Gen (Chen *et al*. 2020) and CMN (Chen *et al*. 2021) across all evaluation metrics. It achieves the lowest proportions of significant errors in all categories (10%–24%), while attaining the highest no-error rates, exceeding 70% in several categories, indicating superior clinical accuracy. Furthermore, 68% of EVOKE-generated reports are ranked as best overall, substantially surpassing R2Gen (22%) and CMN (26%). These results indicate that EVOKE produces reports with fewer clinically significant errors and higher overall quality, highlighting its effectiveness and reliability for automated medical report generation.

**Table 7:** Human evaluation results, including severity assessment across four error types (*SE*: significant error, IE: insignificant error, NE: no error) and overall ranking of candidate reports (1: best, 3: worst).

Model	**(I)**			**(II)**			**(III)**			**(IV)**			**Overall Rank**		
	**SE** ↓	**IE** ↓	**NE** ↑	**SE** ↓	**IE** ↓	**NE** ↑	**SE** ↓	**IE** ↓	**NE** ↑	**SE** ↓	**IE** ↓	**NE** ↑	Best	Moderate	Worse
R2Gen	24%	18%	58%	54%	**38%**	8%	24%	**2%**	74%	32%	32%	36%	22%	46%	32%
CMN	26%	24%	50%	34%	46%	20%	22%	12%	66%	42%	**28%**	30%	26%	48%	26%
**EVOKE**	**16%**	**14%**	**70%**	**24%**	44%	**32%**	**10%**	14%	**76%**	**12%**	34%	**54%**	**68%**	18%	**14%**

**Best values** are highlighted in **bold**.

## 4 Conclusion

In this paper, we presented EVOKE, a novel framework for chest X-ray report generation that jointly leverages multi-view images and patient-specific knowledge (i.e. *indications*). To enhance visual representation learning, we introduced a multi-view contrastive learning method that captures semantic correspondences both across radiographs within a study and between images and reports, effectively handling variable numbers of views. We further proposed a knowledge-guided report generation scheme that flexibly incorporates *indications*, improving clinical contextual reasoning. EVOKE is robust to diverse input settings—including single-view, multi-view, and partial-context scenarios—reflecting the heterogeneity of real-world clinical data. Extensive experiments on MIMIC-CXR, MIMIC-ABN, Multi-view CXR, and Two-view CXR datasets demonstrated consistent gains in both clinical accuracy and language quality. Future work will explore more fine-grained cross-modal alignment strategies (Huang *et al*. 2022, Yuan *et al*. 2023, Cao *et al*. 2024) to further narrow the gap between radiographs and their corresponding reports.

## Author contributions

Qiguang Miao (Conceptualization [Lead], Funding acquisition [Lead], Methodology [Lead], Project administration [Equal], Supervision [Lead], Writing—original draft [Equal], Writing—review & editing [Lead]), Kang Liu (Conceptualization [Lead], Investigation [Equal], Methodology [Lead], Software [Lead], Visualization [Equal], Writing—original draft [Lead], Writing—review & editing [Lead]), Zhuoqi Ma (Conceptualization [Equal], Methodology [Equal], Project administration [Lead], Supervision [Lead], Writing—original draft [Lead], Writing—review & editing [Lead]), Yunan Li (Methodology [Equal], Writing—original draft [Equal], Writing—review & editing [Equal]), Xiaolu Kang (Data curation [Equal], Investigation [Equal], Visualization [Equal]), Ruixuan Liu (Data curation [Lead], Writing—review & editing [Equal]), Tianyi Liu (Data curation [Lead], Writing—review & editing [Equal]), and Kun Xie (Resources [Equal], Writing—original draft [Equal], Writing—review & editing [Equal])

## Supplementary Material

btag566_Supplementary_Data

## Data Availability

The MIMIC-CXR dataset is available through PhysioNet under credentialed access at https://physionet.org/.
