# Supplementary material for: Patient-specific multimodal learning with multi-view contrastive alignment for chest X-ray report generation
Source: Bioinformatics. 2026 Jul 28;42(8):btag566. doi: 10.1093/bioinformatics/btag566 (PMC13472728; doi:10.1093/bioinformatics/btag566)
Supplement: Supplementary file 1 [file btag566_supplementary_data.pdf]

Patient-Specific Multimodal Learning with Multi-View Contrastive Alignment for Chest X-ray Report Generation

Supplementary Material

Additional Results

Implementation Details

1) MIMIC-CXR: In stage 1, we train the model for 50 epochs using the AdamW optimizer with a learning rate of 5e-5. In stage 2, we use the RAdam optimizer for 50 epochs, with learning rates of 5e-6 for the pre-trained model and 5e-5 for other parameters. **2) MIMIC-ABN** and **Multi-view CXR**: As most images in these datasets are derived from MIMIC-CXR (Johnson et al., 2019), Stage 1 is skipped, and the pre-trained model from MIMIC-CXR is fine-tuned using the RAdam optimizer for 50 epochs. Learning rates are set to 5e-6 for MIMIC-ABN (Ni et al., 2020) and 5e-7 for Multi-view CXR. For fairness, all baselines are evaluated using models pre-trained on MIMIC-CXR and fine-tuned before assessment. **3) Two-view CXR**: The same optimizer, learning rate, and epoch settings as MIMIC-CXR are applied. Peer methods are implemented according to their official code, employing concatenation for visual feature fusion. **4) Common settings**: For EVOKE with a resolution of 224×224, radiograph pre-processing follows R2Gen (Chen et al., 2020). For 384×384 resolution, images are resized to 448×448, randomly cropped to 384×384, rotated by 5 degrees, and normalized. Stage 1 and Stage 2 of our framework comprise 242M and 362M parameters, respectively. Experiments are conducted at an input resolution of 224×224 using an NVIDIA 3090 GPU (24GB), while those at a 384×384 resolution are run on an NVIDIA A100 GPU (48GB).

Clinical Accuracy of 14 Observations

Each report is automatically annotated with 14 observations using the CheXpert labeler (Smit et al., 2020). The *No Findings* observation includes only *present* and *absent* states, while the remaining observations additionally allow for *uncertain* and *not-mentioned* states. Following the default configuration of the f1chexbert library (Smit et al., 2020), we treat *not-mentioned* and *absent* states as negative, and all other statuses as positive for binary classification.

Evaluation via Large Language Models

We evaluate the clinical quality of generated reports using GREEN (Ostmeier et al., 2024), a fine-tuned LLaMA-2 model detecting clinically significant errors. The GREEN score is computed as:

$$\text{GREEN} = \frac{\#\text{Matched Findings}}{\#\text{Matched Findings} + \sum_{i=(a)}^{(f)} \#\text{Error}_i}, \quad (1)$$

where $\#\text{Error}_i$ counts the ith error type. GREEN categorizes clinically significant errors into six types: (a) False report of a finding in the candidate; (b) Missing a finding present in the reference; (c) Misidentification of a finding's anatomic location/position; (d) Misassessment of the severity of a finding; (e) Mentioning a comparison that isn't in the reference; (f) Omitting a comparison detailing a change from a prior study.

Qualitative Analysis of Multi-view Radiograph Report Generation

Fig. 1 presents representative examples of reports generated by EVOKE from chest radiographs with different numbers of views. In Case 1, the small bilateral pleural effusions are visible only on the lateral view and therefore cannot be reliably identified from the frontal image alone. Consequently, they are omitted in the single-view report but are correctly described when additional views are incorporated. In Case 2, the frontal projection provides limited characterization of the retrocardiac opacity because of anatomical superimposition, causing the hiatal hernia to be misidentified as an elevated left hemidiaphragm. The additional lateral view reveals the characteristic retrocardiac air–fluid level and facilitates the correct diagnosis. These examples demonstrate that increasing the number of views provides complementary diagnostic information, allowing EVOKE to resolve ambiguities in individual projections and generate more clinically accurate reports.

REFERENCES

Z. Chen, Y. Song, T.-H. Chang, and X. Wan. Generating radiology reports via memory-driven transformer. In EMNLP, pages 1439–1449, 2020.

A. E. W. Johnson, T. J. Pollard, S. J. Berkowitz, N. R. Greenbaum, M. P. Lungren, C.-y. Deng, R. G. Mark, and S. Horng. Mimic-cxr, a de-identified publicly available database of chest radiographs with free-text reports. Scientific Data, 6(1), Dec. 2019.

J. Ni, C. Hsu, A. Gentili, and J. J. McAuley. Learning visual-semantic embeddings for reporting abnormal findings on chest x-rays. In EMNLP, pages 1954–1960, 2020.

S. Ostmeier, J. Xu, Z. Chen, M. Varma, L. Blankemeier, C. Bluethgen, A. E. M. Md, M. Moseley, C. Langlotz, A. S. Chaudhari, and J.-B. Delbrouck. GREEN: Generative radiology report evaluation and error notation. In EMNLP, pages 374–390, Nov. 2024.

A. Smit, S. Jain, P. Rajpurkar, A. Pareek, A. Ng, and M. Lungren. Combining automatic labelers and expert annotations for accurate radiology report labeling using bert. In EMNLP, 2020.

Table 1 Dataset statistics. "#Img" and "#Rpt" denote the numbers of radiographs and reports, respectively; "%Ind" indicates the proportion of *indications*. The sample size equals "#Img" for each dataset.

Split	MIMIC-CXR			MIMIC-ABN			Multi-view CXR			Two-view CXR		
	#Img	#Rpt	%Ind	#Img	#Rpt	%Ind	#Img	#Rpt	%Ind	#Img	#Rpt	%Ind
Train	269,239	150,957	66.4	69,526	34,763	64.6	220,978	100,505	67.1	165,056	82,528	67.9
Val	2,113	1,182	65.4	526	263	62.7	2,299	1,057	71.2	1,778	889	72.1
Test	3,852	2,343	57.3	756	378	56.3	3,947	1,805	67.6	3,000	1,500	68.9

Table 2 Clinical accuracy of our EVOKE on the MIMIC-CXR and Two-view CXR datasets with a resolution of 224×224. ECM stands for *Enlarged Cardiomediastinum*. P, R, and F_1 denote the precision, recall, and F_1 score, respectively.

Observation	MIMIC-CXR							Two-view CXR						
	%	R2Gen			EVOKE (Ours)			%	R2Gen			EVOKE (Ours)		
		P	R	F_1	P	R	F_1		P	R	F_1	P	R	F_1
ECM	10.2	0.377	**0.353**	0.365	**0.396**	0.345	**0.369**	9.8	**0.371**	0.280	0.319	0.351	**0.300**	**0.324**
Cardiomegaly	14.7	0.567	0.347	0.431	**0.628**	**0.566**	**0.595**	14.1	**0.590**	0.409	0.483	0.578	**0.567**	**0.573**
Lung Opacity	13.3	0.457	0.231	0.307	**0.578**	**0.352**	**0.437**	13.1	**0.614**	0.069	0.124	0.567	**0.335**	**0.421**
Lung Lesion	2.6	**0.481**	**0.046**	**0.084**	0.217	0.036	0.061	2.8	**0.500**	0.012	0.023	0.357	**0.060**	**0.102**
Edema	8.3	0.445	0.254	0.323	**0.514**	**0.447**	**0.478**	5.8	**0.523**	0.259	0.346	0.475	**0.333**	**0.392**
Consolidation	3.3	0.188	0.096	0.127	**0.240**	**0.154**	**0.188**	3.5	**0.300**	0.115	0.167	0.279	**0.163**	**0.206**
Pneumonia	4.6	0.238	**0.204**	**0.220**	**0.264**	0.186	0.218	3.7	0.276	0.071	0.113	**0.349**	**0.268**	**0.303**
Atelectasis	11.1	0.421	0.249	0.313	**0.496**	**0.519**	**0.507**	10.2	0.463	0.248	0.323	**0.483**	**0.464**	**0.473**
Pneumothorax	1.0	**0.333**	0.029	0.054	0.291	**0.157**	**0.204**	0.8	0.000	0.000	0.000	**0.222**	**0.087**	**0.125**
Pleural Effusion	12.5	**0.804**	0.196	0.316	0.716	**0.669**	**0.691**	10.5	**0.787**	0.375	0.508	0.709	**0.667**	**0.687**
Pleural Other	1.7	0.000	0.000	0.000	**0.217**	**0.083**	**0.120**	1.9	**0.500**	0.018	0.034	0.091	**0.036**	**0.051**
Fracture	2.0	0.000	0.000	0.000	**0.071**	**0.014**	**0.023**	2.3	0.000	0.000	0.000	**0.188**	**0.043**	**0.070**
Support Devices	12.5	0.723	0.489	0.583	**0.755**	**0.713**	**0.734**	9.5	0.706	0.488	0.577	**0.722**	**0.646**	**0.681**
No Finding	2.2	0.130	0.626	0.215	**0.223**	**0.660**	**0.333**	12.0	0.387	**0.950**	0.550	**0.547**	0.813	**0.654**
micro avg	-	0.432	0.280	0.340	**0.538**	**0.465**	**0.499**	-	0.484	0.341	0.400	**0.539**	**0.469**	**0.501**
macro avg	-	0.369	0.223	0.238	**0.400**	**0.350**	**0.354**	-	**0.430**	0.235	0.255	0.423	**0.342**	**0.362**

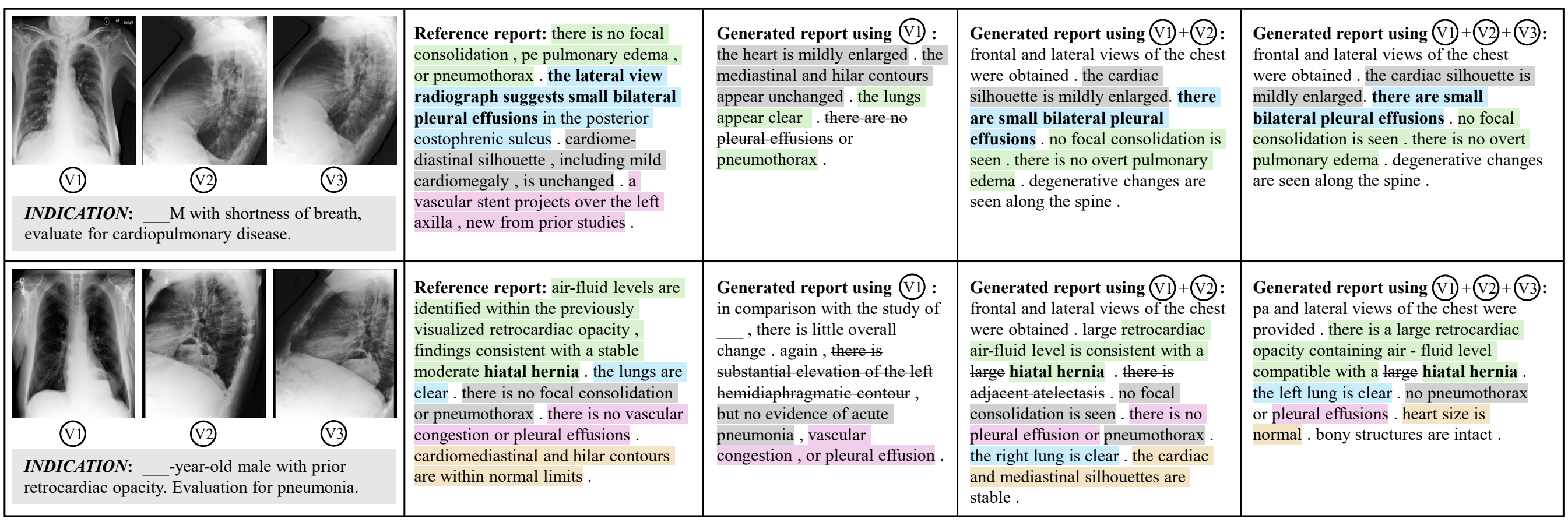

Figure 1 Representative examples comparing EVOKE-generated reports from one-, two-, and three-view chest radiographs. Reference sentences are color-coded, and the corresponding generated sentences are highlighted using the same colors. **Bold** text denotes findings that require additional views for reliable identification, whereas ~~strikethrough text~~ indicates clinically significant errors.

Alt-text: Representative cases demonstrating improved chest X-ray report generation with multi-view radiographs.
